# Structure and Receptor binding properties of a pandemic H1N1 virus hemagglutinin

**DOI:** 10.1371/currents.RRN1152

**Published:** 2010-03-23

**Authors:** Hua Yang, Paul Carney, James Stevens

**Affiliations:** Centers for Disease Control and Prevention

## Abstract

The 3D-structure of the major surface viral antigen from the recent H1N1 pandemic influenza virus (A/Darwin/2001/2009) was determined to 2.8 Å resolution. The structure was used to analyze changes in the HA that have emerged during the first 11 months of the pandemic and have raised public health concerns. Receptor binding properties of this protein reveals a strict preference for human-type receptors.

## Introduction

        The first influenza pandemic of the new century emerged in April 2009, when a new H1N1 influenza virus (H1N1pdm), found in patients in Mexico and the United States, spread rapidly across the world by human-to-human transmission, resulting in the World Health Organization declaring a global pandemic on June 11^th^ 2009 [1]. The pandemic H1N1 virus (2009 H1N1) was unique in that it had a gene constellation from both North American and Eurasian swine lineages that had not been isolated previously in either swine or human populations [2]. Phylogenetic and antigenic analysis of the hemagglutinin (HA) gene revealed it to be distinct from seasonal human H1N1 viruses but more similar to the classical North American swine lineage.

        Ten months after the first viruses were isolated, the virus is still antigenically homogeneous [3]. However, as the HA continues to circulate in the human population, its HA antigenic sites will continue to be targeted by antibody-mediated selection pressure. Therefore it is important from a public health perspective to structurally characterize the hemagglutinin so that the research community has a template with which to visualize any changes affecting antigenicity or virulence that may emerge as this virus evolves. To this end, we have cloned, expressed and solved the structure of a pandemic H1 hemagglutinin by x-ray crystallography. The structure was used to analyze amino acid substitutions in the HA that have raised some concern during the last 11 months of global surveillance activities. The same protein was analyzed by glycan microarray and compared to seasonal and other pandemic variants. Results reveal a strict human-like receptor specificity.

## Materials and Methods

*Recombinant HA cloning and expression:* Utilizing a similar cloning strategy from previous studies [4] [5] [6], the HA ectodomain of the 2009 H1N1 pandemic influenza virus, A/Texas/05/2009 (Accession: FJ966959) was codon optimized, synthesized and cloned into the baculovirus transfer vector, pAcGP67-A (BD Biosciences, San Jose, CA) by Geneart AG, Germany. Constructs containing Ohio/7/2009 (Accession: FJ969535), A/Utah/20/2009 (Gisaid Accession: EPI217204) and A/Darwin/2001/2009 (Accession: GQ243757) were generated by mutagenesis of the A/Texas/05/2009 clone (A/Ohio/7/2009:Ser203Thr/Val411Ile; A/Darwin2001/2009:Ser203Thr/Arg205Lys/Val411Ile; A/Utah/20/2009:Asn156Asp/Gln293His) using the QuikChange Multi Site-Directed Mutagenesis Kit (Stratagene, CA). Seasonal H1N1 HA constructs were cloned into the baculovirus transfer vector, pAcGP67-A (BD Biosciences, San Jose, CA). Transfection and virus amplification were carried out as described previously [4] [5] [6]. Protein expressed from Trichoplusia ni (Hi5) cells (Invitrogen, Carlsbad, CA) in 10-stack CellSTACK™ culture chambers (Corning Inc., Corning, NY) was recovered from the culture supernatant and purified by metal affinity chromatography, subjected to thrombin cleavage and gel filtration chromatography [7]. Purified monomeric protein was buffer exchanged into 10 mM Tris-HCl, 50 mM NaCl, pH 8.0 and concentrated to 7.8 mg/ml for crystallization trials. At this stage, the protein sample still contained the additional plasmid-encoded residues at both the N (ADPG) and C terminus (SGRLVPR).

*Crystallization and data collection:* Initial crystallization trials were set up using a Topaz^TM^ Free Interface Diffusion (FID) Crystallizer system (Fluidigm Corporation, San Francisco, CA). Crystals were observed in conditions containing various molecular weights of PEG polymer. Following optimization, diffraction quality crystals for Darwin09 were obtained at 20 ºC using a modified method for ‘microbath under oil’ [8], by mixing the protein with reservoir solution containing 22% PEG2000MME, 0.1M HEPES at pH 7.5. Crystals were flash-cooled at 100K, data was collected at the Advanced Photon Source (APS) beamline 22-BM at 100K and processed with the DENZO-SACLEPACK suite [9]. The data were indexed in spacegroup P1 with unit cell dimensions a=73.98Å, b=109.71Å, c=129.90Å; α=86.25°, β=74.68°, ϒ=75.10°.  Statistics for data collection are presented in Table 1.

*Structure determination and refinement: *The structure of Darwin09 was determined by molecular replacement with Phaser [10] using the HA structure from A/Japan/305/1957, pdb:3KU3 [11] (HA1, 55% identity; HA2, 82% identity) as the search model. Six hemagglutinin monomers making one non-crystallographic trimer, related by a non-crystallographic 3-fold and three monomers that form one-third and two-thirds of two crystallographic trimers, occupy the asymmetric unit with an estimated solvent content of 55% based on a Matthews’ coefficient (Vm) of 2.75 Å^3^/Da. Rigid body refinement of the trimer led to an overall R/Rfree of 48.1%/48.6 %. The model was then “mutated” to the correct sequence and rebuilt by Coot [12], then the protein structures were refined with REFMAC [13] using TLS refinement [14]. The final models were assessed using MolProbity [15]. Statistics for data processing and refinement are presented in Table 1.

**Table 1**            Data collection and refinement statistics. 

**Table:** 

**Data collection**	**Darwin09**
Space group	P1
Cell dimensions	a=73.98Å, b=109.71Å, c=129.90Å α=86.25°, β=74.68°, ϒ=75.10°
Resolution (Å)	50-2.8 (2.90-2.80)*
R_sym _	7.6 (44.2)
<I/σ>	9.7 (1.6)
Completeness (%)	98.1 (95.7)
Redundancy	2.0 (1.9)
**Refinement**	
Resolution (Å)	50-2.8 (2.87-2.80)
No. of reflections (total)	87344
No. of reflections (test)	4608
R_work_/ R_free_	23.1/25.6
No. of atoms	23552
r.m.s.d.- bond length (Å)	0.016
r.m.s.d.- bond angle (°)	1.701
**MolProbity^#^ scores **	
Favored (%)	95.3
Allowed (%)	99.7
Outliers (%) _(No. of residues)_	0.3 _(9/2934)_

* Numbers in parentheses refer to the highest resolution shell. # Reference [15]

*Glycan microarray* *analysis:* Microarray printing and recombinant HA analyses have been described previously [6] [16]. Imprinted slides produced specifically for influenza research for the CDC using the CFG glycan library (CDC version 1 slides; see Table 2 for glycans used in these experiments) were used.

**Table 2** **Glycans covalently attached on the glycan microarray**. Different categories of glycans on the array are color-coded in column 1 as follows: No color, sialic acid; blue, α2-3 sialosides; red, α2-6 sialosides, violet, mixed α2-3/ α2-6 biantennaries; green, N-glycolylneuraminic acid-containing glycans; brown, α2-8 linked sialosides; pink, b2-6 linked as well as 9-O-acetylated sialic acids; grey, asialo glycans. 

**Table:** 

Chart #	Structure	Description
1	α-Neu5Ac	α-Neu5Ac
2	α-Neu5Ac	α-Neu5Ac
3	b-Neu5Ac	β-Neu5Ac
4	Neu5Acα2-3(6-O-Su)Galβ1-4(Fucα1-3)GlcNAcβ	α2-3 so4
5	Neu5Acα2-3Galβ1-3[6OSO3]GalNAcα	α2-3 so4
6	Neu5Acα2-3Galβ1-4[6OSO3]GlcNAcβ	α2-3 so4
7	Neu5Acα2-3Galβ1-4(Fucα1-3)(6OSO3)GlcNAcβ	α2-3 so4
8	Neu5Acα2-3Galβ1-3(6OSO3)GlcNAc	α2-3 so4
9	Neu5Acα2-3Galβ1-3(Neu5Acα2-3Galβ1-4)GlcNAcβ	di-sialoside
10	Neu5Acα2-3Galβ1-3(Neu5Acα2-3Galβ1-4GlcNAcβ1-6)GalNAcβ	di-sialoside
11	Neu5Acα2-3Galβ1-4GlcNAcβ1-2Manα1-3(Neu5Acα2-3Galβ1-4GlcNAcβ1­2Manα1-6)Manβ1-4GlcNAcβ1-4GlcNAcβ	α2-3 biantennary
12	Neu5Acα2-3Galβ	α2-3
13	Neu5Acα2-3GalNAcα	α2-3
14	Neu5Acα2-3Galβ1-3GalNAcα	α2-3
15	Neu5Acα2-3Galβ1-3GlcNAcβ	α2-3
16	Neu5Acα2-3Galβ1-3GlcNAcβ	α2-3
17	Neu5Acα2-3Galβ1-4Glcβ	α2-3
18	Neu5Acα2-3Galβ1-4Glcβ	α2-3
19	Neu5Acα2-3Galβ1-4GlcNAcβ	α2-3
20	Neu5Acα2-3Galβ1-4GlcNAcβ	α2-3
21	Neu5Acα2-3GalNAcβ1-4GlcNAcβ	α2-3
22	Neu5Acα2-3Galβ1-4GlcNAcβ1-3Galβ1-4GlcNAcβ	α2-3
23	Neu5Acα2-3Galβ1-3GlcNAcβ1-3Galβ1-3GlcNAcβ	α2-3
24	Neu5Acα2-3Galβ1-4GlcNAcβ1-3Galβ1-4GlcNAcβ1-3Galβ1-4GlcNAcβ	α2-3
25	Neu5Acα2-3Galβ1-4GlcNAcβ1-3Galβ1-3GlcNAcβ	α2-3
26	Neu5Acα2-3Galβ1-3GalNAcα	α2-3
27	Galβ1-3(Neu5Acα2-3Galβ1-4(Fucα1-3)GlcNAcβ1-6)GalNAcβ	α2-3 fucosylated
28	Neu5Acα2-3Galβ1-3(Fucα1-4)GlcNAcβ	α2-3 fucosylated
29	Neu5Acα2-3Galβ1-4(Fucα1-3)GlcNAcβ	α2-3 fucosylated
30	Neu5Acα2-3Galβ1-4(Fucα1-3)GlcNAcβ	α2-3 fucosylated
31	Neu5Acα2-3Galβ1-4(Fucα1-3)GlcNAcβ1-3Galβ	α2-3 fucosylated
32	Neu5Acα2-3Galβ1-4(Fucα1-3)GlcNAcβ1-3Galβ1-4GlcNAcβ	α2-3 fucosylated
33	Neu5Acα2-3Galβ1-4(Fucα1-3)GlcNAcβ1-3Galβ1-4(Fucα1-3)GlcNAcβ1­3Galβ1-4(Fucα1-3)GlcNAcβ	α2-3 fucosylated
34	Neu5Acα2-3Galβ1-4GlcNAcβ1-3Galβ1-4(Fucα1-3)GlcNAc	α2-3 fucosylated
35	Neu5Acα2-3(GalNAcβ1-4)Galβ1-4GlcNAcβ	α2-3 internal
36	Neu5Acα2-3(GalNAcβ1-4)Galβ1-4GlcNAcβ	α2-3 internal
37	Neu5Acα2-3(GalNAcβ1-4)Galβ1-4Glcβ	α2-3 internal
38	Galβ1-3GalNAcβ1-4(Neu5Acα2-3)Galβ1-4Glcβ	α2-3 internal
39	Fucα1-2Galβ1-3GalNAcβ1-4(Neu5Acα2-3)Galβ1-4Glcβ	α2-3 internal
40	Fucα1-2Galβ1-3GalNAcβ1-4(Neu5Acα2-3)Galβ1-4Glcβ	α2-3 internal
41	Neu5Acα2-6Galβ1-4[6OSO3]GlcNAcβ	α2-6 so4
42	Galβ1-4GlcNAcβ1-2Manα1-3(Neu5Acα2-6Galβ1-4GlcNAcβ1-2Manα1­6)Manβ1-4GlcNAcβ1-4GlcNAcβ	α2-6 branched
43	GlcNAcβ1-2Manα1-3(Neu5Acα2-6Galβ1-4GlcNAcβ1-2Manα1-6)Manβ1­4GlcNAcβ1-4GlcNAcβ	α2-6 branched
44	Galβ1-4GlcNAcβ1-2Manα1-3(Neu5Acα2-6Galβ1-4GlcNAcβ1-2Manα1­6)Manβ1-4GlcNAcβ1-4GlcNAcβ	α2-6 branched
45	Neu5Acα2-6Galβ1-4GlcNAcβ1-2Manα1-3(GlcNAcβ1-2Manα1-6)Manβ1­4GlcNAcβ1-4GlcNAcβ	α2-6 branched
46	Neu5Acα2-6Galβ1-4GlcNAcβ1-2Manα1-3(Neu5Acα2-6Galβ1-4GlcNAcβ1­2Manα1-6)Manβ1-4GlcNAcβ1-4GlcNAcβ	α2-6 biantenary
47	Neu5Acα2-6Galβ1-4GlcNAcβ1-2Manα1-3(Neu5Acα2-6Galβ1-4GlcNAcβ1­2Manα1-6)Manβ1-4GlcNAcβ1-4GlcNAcβ	α2-6 biantenary
48	Neu5Acα2-6Galβ1-4GlcNAcβ1-2Manα1-3(Neu5Acα2-6Galβ1-4GlcNAcβ1­2Manα1-6)Manβ1-4GlcNAcβ1-4GlcNAcβ	α2-6 biantenary
49	Neu5Acα2-6Galβ1-4GlcNAcβ1-2Manα1-3(Galβ1-4GlcNAcβ1-2Manα1­6)Manβ1-4GlcNAcβ1-4GlcNAcβ	α2-6 biantenary
50	Neu5Acα2-6GalNAcα	α2-6
51	Neu5Acα2-6Galβ	α2-6
52	Neu5Acα2-6Galβ1-4Glcβ	α2-6
53	Neu5Acα2-6Galβ1-4GlcNAcβ	α2-6
54	Neu5Acα2-6Galβ1-4GlcNAcβ	α2-6
55	Neu5Acα2-6GalNAcβ1-4GlcNAcβ	α2-6
56	Neu5Acα2-6Galβ1-4GlcNAcβ1-3Galβ1-4GlcNAcβ	α2-6
57	Neu5Acα2-6Galβ1-4GlcNAcβ1-3Galβ1-4(Fucα1-3)GlcNAcβ1-3Galβ1­4(Fucα1-3)GlcNAcβ	α2-6 + fucosylation
58	Galβ1-3(Neu5Acα2-6)GlcNAcβ1-3Galβ1-4Glcβ	α2-6 internal
59	Galβ1-3(Neu5Acα2-6)GalNAcα	α2-6 internal
60	Neu5Acα2-3Galβ1-4GlcNAcβ1-2Manα1-3(Neu5Acα2-6Galβ1-4GlcNAcβ1­2Manα1-6)Manβ1-4GlcNAcβ1-4GlcNAcβ	α2-3/6 biantennary
61	Neu5Acα2-6Galβ1-4GlcNAcβ1-2Manα1-3(Neu5Acα2-3Galβ1-4GlcNAcβ1­2Manα1-6)Manβ1-4GlcNAcβ1-4GlcNAcβ	α2-3/6 biantennary
62	Neu5Acα2-3Galβ1-3(Neu5Acα2-6)GalNAc	α2-3/6 disialoside
63	Neu5Acα2-3Galβ1-3(Neu5Acα2-6)GalNAcα	α2-3/6 disialoside
64	Neu5Acα2-3(Neu5Acα2-6)GalNAcα	α2-3/6 disialoside
65	Neu5Gcα	Neu5Gc α
66	Neu5Gcα2-3Galβ1-3(Fucα1-4)GlcNAcβ	Neu5Gc α2-3
67	Neu5Gca2-3Galβ1-3GlcNAcβ	Neu5Gc α2-3
68	Neu5Gcα2-3Galβ1-4(Fucα1-3)GlcNAcβ	Neu5Gc α2-3
69	Neu5Gcα2-3Galβ1-4GlcNAcβ	Neu5Gc α2-3
70	Neu5Gcα2-3Galβ1-4Glcβ	Neu5Gc α2-3
71	Neu5Gcα2-6GalNAcα	Neu5Gc α2-6
72	Neu5Gcα2-6Galβ1-4GlcNAcβ	Neu5Gc α2-6
73	Neu5Acα2-8Neu5Acα	Neu5Ac α2-8
74	Neu5Acα2-8Neu5Acα2-8Neu5Acα	Neu5Ac α2-8
75	Neu5Acα2-8Neu5Acα2-3(GalNAcβ1-4)Galβ1-4Glcβ	Neu5Ac α2-8 α2-3
76	Neu5Acα2-8Neu5Acα2-3Galβ1-4Glcβ	Neu5Ac α2-8 α2-3
77	Neu5Acα2-8Neu5Acα2-8Neu5Acα2-3(GalNAcβ1-4)Galβ1-4Glcβ	Neu5Ac α2-8 α2-8 α2-3
78	Neu5Acα2-8Neu5Acα2-8Neu5Acα2-3Galβ1-4Glcβ	Neu5Ac α2-8 α2-8 α2-3
79	Neu5Acα2-8Neu5Acα	Neu5Ac α2-8
80	Neu5Acα2-8Neu5Acβ	Neu5Ac α2-8
81	Neu5Acα2-8Neu5Acα2-8Neu5Acβ	Neu5Ac α2-8 α2-8
82	Neu5Acβ2-6GalNAcα	β2-6
83	Neu5Acβ2-6Galβ1-4GlcNAcβ	β2-6
84	Neu5Gcβ2-6Galβ1-4GlcNAc	β2-6
85	Galβ1-3(Neu5Acβ2-6)GalNAcα	β2-6
86	9NAcNeu5Aca	9NAcNeu5
87	9NAcNeu5Acα2-6Galβ1-4GlcNAcβ	9NAcNeu5
88	Galβ1-4GlcNAcβ1-3Galβ1-4GlcNAcβ1-3Galβ1-4GlcNAcβ	asialo
89	Galβ1-3GlcNAcβ1-3Galβ1-3GlcNAcβ	asialo
90	Fucα1-2Galβ1-3GlcNAcβ1-3Galβ1-4Glcβ	asialo
91	Fucα1-2Galβ1-4(Fucα1-3)GlcNAcβ1-3Galβ1-4(Fucα1-3)GlcNAcβ	asialo
92	GalNAcα1-3(Fucα1-2)Galβ1-3GlcNAcβ	asialo
93	GalNAcα1-3(Fucα1-2)Galβ1-4GlcNAcβ	asialo
94	Galα1-3(Fucα1-2)Galβ1-3GlcNAcβ	asialo
95	Galα1-3(Fucα1-2)Galβ1-4(Fucα1-3)GlcNAcβ	asialo
96	Galβ1-3GalNAc	asialo

       

            ***Key*** ***:***     

            Neu5Ac = Sialic acid

            Neu5Gc = N-glycolylneuraminic acid

            OSO_3_ = sulfate; Gal = galactose

            Fuc = fucose

            GlcNAc = N-Acetyl-D-glucosamine

            GalNAc = N-acetyl-D-galactosamine

            Glc = D-glucose

            Man = D-mannose

            9NAc = 9-*O*-acetyl 

## Results and Discussion

        Expression and purification Recombinant HA protein from A/Darwin/2001/2009 (H1N1) (Darwin09) virus was expressed in a baculovirus expression system utilizing a thrombin site at the C-terminus of Darwin09 followed by a trimerizing sequence (foldon) from the bacteriophage T4 fibritin for generating functional trimers [17], and a His-Tag to aid purification. Although protein was expressed as a trimer, only monomers were purified by gel filtration after foldon removal by the thrombin cleavage step. However, these monomers were stable, the protein stock maintained its monomeric state even after 8 weeks storage at 4 °C (confirmed by dynamic light scattering analysis). However, monomers were still able to reform trimers in the crystal as evidenced by the structure reported here.

**Figure:**
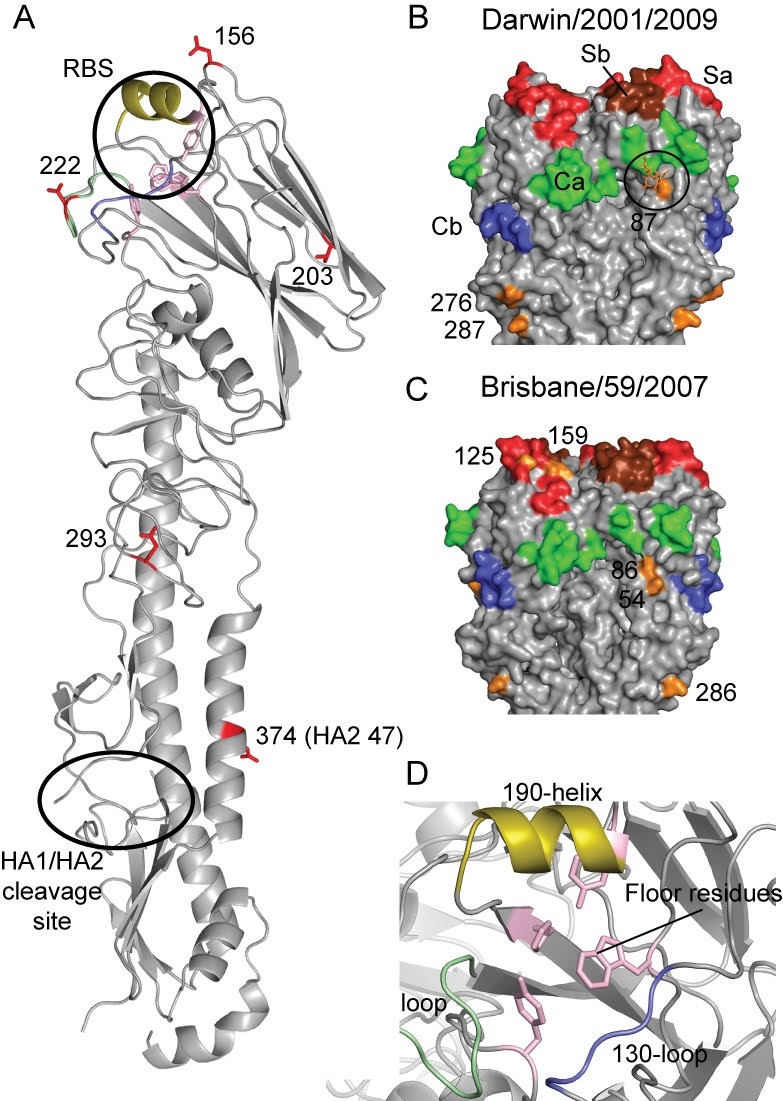
**Figure 1** **Structural overview of the** **Darwin09 HA monomer.** (A)One monomer is shown with the location of the receptor-binding site (RBS) and the HA1/HA2 cleavage site circled. The positions of residues discussed in the text are highlighted in red **(**B) H1 HA antigenic sites, Ca, Cb, Sa and Sb are mapped onto a surface representation of the HA1 domain of the Darwin trimer with positions of nearby potential glycosylation sites colored orange. (C) For comparison, a model of the last H1N1 seasonal vaccine component, A/Brisbane/59/2009 was generated by homology modeling [18] . (D) RBS of Darwin09 with the three structural elements comprising this binding site, the 130-loop, 220-loop and the 190-helix, colored light blue, light green and olive, respectively. All the figures were generated and rendered with the use of MacPyMOL [19].

        Overall Structure By using x-ray crystallography, the structure of pandemic H1N1 HA from the Darwin09 virus was determined to 2.8 Å resolution (Table 1). The overall structure of Darwin09 is similar to other reported HA structures with a globular head containing the RBS and vestigial esterase domain, and a membrane proximal domain with its distinctive, central helical stalk and HA1/HA2 cleavage site (Figure 1A). We selected representative HAs from human pandemic subtypes for structural analysis. Darwin09 HA was found to be structurally very similar to the 1918-pandemic HA and the pandemic potential H5N1 HA in comparisons. Although closely related to the HA2 domains of the other swine H1, H2 and H3 subtypes in the analysis, the HA1 domains were more divergent (Table 3).

**Table 3.** **Comparison of r.m.s.d. (Å) for HA1 and HA2 domains.** For analyzing differences in the overall structure, r.m.s.d. values were calculated between monomers or domains of different pandemic and pandemic potential HA’s, after the Ca atoms of the HA2 domains were superposed by sequence and structural alignment onto the equivalent domains of Darwin09. 

**Table:** 

**S** **ubtype**	**PDB entry**	**HA1 Domain**	**HA2 Domain**
1918-Hu-H1N1 South Carolina/1/18	1RD8	0.57	1.10
1930-Swine-H1N1 A/swine/Iowa/30,	1RUY	2.38	1.33
1957-Hu-H2N2 A/Japan/305/57	3KU5	3.23	1.76
1968-Hu-H3N2 A/Hong Kong/1/68	2HMG	7.08	1.86
2004-Hu-H5N1 A/Vietnam/1203/04	2FK0	1.52	0.88

        Although six asparagine-linked glycosylation sequons are present in the Darwin09 HA monomer, interpretable electron density was observed at only 3 sites in HA1, Asn23, Asn87 and Asn276. At these sites, only one or two N-acetyl glucosamines could be interpreted. Compared to recent seasonal HAs, potential glycosylation sites in the HA1 of the pandemic HA are in comparable positions (Figure 1B and C). Position 87 in the pandemic HA is also a glycosylation site in seasonal HAs and has been a conserved feature since 1918 [7]. On recent H1 HAs, a second site, at Asn54, is in very close proximity to Asn87 and it is not known whether both sites are occupied. Similarly, the pandemic HA also has two potential glycosylation sites at positions 276 and 286, at the bottom of the HA1 that are close together. However, no conclusions can be made from this structure with respect to double occupancy at these positions since density was only observed at position 276 in two of the six chains in the asymmetric unit.

        The receptor binding domain The receptor-binding site (RBS) is at the membrane distal end of each HA monomer and its specificity for sialic acid and the nature of its linkage to a vicinal galactose residue determines host range-restriction. As for other HA structures, the Darwin09 RBS is composed of three structural elements: a 190-helix (residues 184-191), a 220-loop (residues 218-225), and a 130-loop (residues 131-135), while other highly conserved residues: Tyr91, Trp150, His180, and Tyr192 form the base of the pocket (Figure 1D).

        Interestingly, previous published research highlighted dual receptor specificity for the early pandemic viruses [20]. Using carbohydrate microarray analysis, the authors observed mixed a2-3/ α2-6 receptor specificity with two pandemic viruses (California/4/2009 and Hamburg/5/2009), while a seasonal H1N1 virus bound exclusively to α2-6-linked sialosides. Using recombinant HA we can also probe these microarray platforms [4] [5] [6]. By pre-complexing trimers using primary and secondary antibodies one can overcome the low affinity of HA for its glycan ligand [21] by increasing the valency. Results using recombinant HA revealed a strict preference for five human-type sialyl-glycans, with no significant binding to avian α2-3 receptor analogs. All pandemic recombinant HAs bound to a α2-6 sialylated tri-N-acetyllactosamine glycan in which the two proximal (reducing end) lactosamines are α1-3 fucosylated (glycan #57 in the Table 2) as well as to a structurally related long linear α2-6 sialylated di-N-acetyllactosamine (Figure 2, glycan #56). These glycans were detected in N-glycans of cultured human bronchial epithelial cells [22]. Two other structurally diverse glycans, a α2-6 sialylated-sulfated N-acetyllactosamine structure (glycan #41) and the α2-6 sialylated LacNAc (glycans #53 & 54) were also recognized by these HAs. In addition, the proteins in this study bound weakly to α2-6 sialylated bi-antennary glycans (glycans #46-48), which are typically found on membrane glycoproteins [23]. These results were comparable to the two seasonal HAs used in the analysis (A/Solomon Islands/3/2006 and A/Brisbane/59/2007 are the two H1N1 components of the 2007-2008, 2008-2009 and 2009-2010 trivalent vaccine) although good binding to the α2-6 sialylated bi-antennary glycans (glycans #46-48) was observed for the Solomon Islands/3/2006 recombinant HA. Thus, these pandemic viruses bind to human type receptors as shown and postulated by previous reports [24] [25]. This strict specificity is in contrast to the Childs et al report [20]. However, these differences can be attributed to the different platforms used as well as increased valency of the virus, which might enhance binding to weak ligands.

**Figure:**
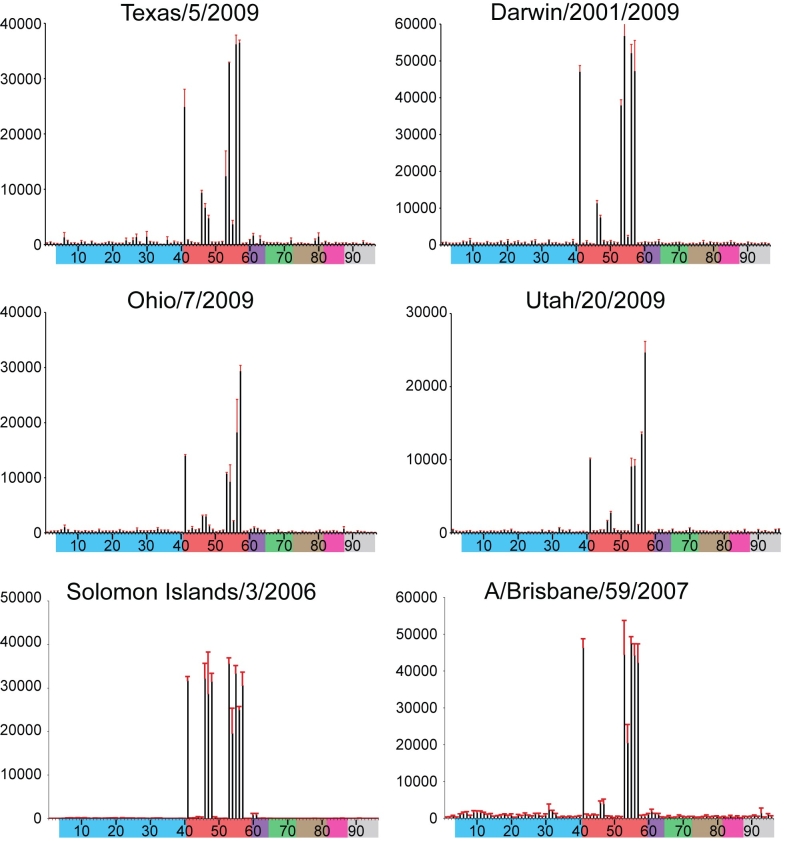
**Figure 2** Glycan microarray analysis of pandemic H1 recombinant HAs. Protein of A/Texas/5/2009, A/Darwin/2001/2009, A/Ohio/7/2009 and A/Utah/20/2009 were analyzed and compared to the recent vaccine candidates from seasonal H1 HAs, A/Solomon Islands/3/2006 and A/Brisbane/59/2007. Colored bars highlight glycans that contain α2-3 SA (blue) and α2-6 SA (red), α2-6/ α2–3-mixed SA (purple), N-glycolyl SA (green), α2-8 SA (brown), b2-6 and 9-O-acetyl SA, and non-SA (grey). Error bars reﬂect the standard deviation in the signal for six independent replicates on the array. Structures of each of the numbered glycans are found in Table 2.

        Genetic and antigenic changes Four antigenic sites for H1N1 virus HAs, have been identified (Ca, Cb, Sa, and Sb) [26] [27]. In Darwin09, with the exception of Ca, all are exposed for antibody recognition. The Ca site is proximal to the oligosaccharide at HA1 Asn87, which may interfere with antibody recognition of this region. In recent seasonal H1 HAs the Sa site (and possibly Sb) looks to be affected by the presence of two glycosylation sites at positions 125 and 159 (Figure 1D). Lack of these sites in the pandemic HA exposes the entire top of the HA1 for targeting by the immune system and this feature may explain why the antibody recall response to the pandemic vaccine in adults was so effective [28].

        Since the pandemic virus first emerged, the majority of viruses have shared a Ser203Thr amino acid change in the HA. This position is near the monomer-monomer interface and the small change in side chain appears not to have had a dramatic effect on the HA structure. Introduction of the extra methylene group in the side chain may help to stabilize the loop region in its surrounding environment (Figure 1A). Currently, two circulating subsets of viruses have amino acid changes, Asp222Glu or Glu374Lys, in the HA. The Asp222Glu mutation is in the receptor-binding site and may modulate which glycans bind to the receptor (Figure 1A). The latter mutation at position 374 is in the HA2 (residue 47) and points into the cavity where the fusion peptide resides in the mature fusion ready form of the HA molecule. Although this mutation may affect stability in this region (Figure 1A), it is also close to a region identified by two recent HA/neutralizing antibody structures which target the stem region of the HA [29] [30]. Little is known about the immune response to this region and whether this mutation is able to modulate antibody binding.

        Other HA mutations have also been observed that affect antigenicity, but most have been sporadic throughout the year, geographically separated or results of egg growth [31]. In particular, changes at positions 153-157 in the HA have been associated with reduced HI titers with ferret antisera to the A/California/7/09 vaccine virus. In most (if not all) cases, these changes have emerged after virus propagation in cell cultures. The structure highlights this region to be a prominent loop on the top left of the receptor binding site and is a component of the Sa (H1) or Site B (H3) antigenic site (Figure 1A and 1B) [27]. In the pandemic H1 HA, this region is exposed to the host immune system and not masked by vicinal glycosylation sites. Although this position is known to affect antigenicity, it does not appear to change receptor binding as shown by the glycan microarray results for A/Utah/20/2009 which has as Asn156Asp change compared to the other pandemic virus HAs analyzed (Figure 2). Its ability to change easily also highlights this region as a potential ‘hot spot’ for future mutation as the human population gains immunity and the virus experiences increased pressure to evade the immune response.

        More recently, there has been focus on the possible role of a mutation at position 222 and its role in severe clinical outcome [32] [33]. The Asp222Gly and Asp222Asn single and mixed variants have been found in pandemic viruses as well as direct sequencing from clinical specimens collected throughout the 2009 pandemic from approximately 20 countries, including Norway, Mexico, Ukraine and the USA. As already described above, position 222 resides in the receptor binding site of the HA protein and may possibly influence binding specificity. Indeed, the HA from the previous H1N1 pandemic in 1918 switched from avian to human receptor specificity through mutation at two positions (Glu187Asp and Gly222Asp) [5]. (The pandemic virus HA is also an Asp at position 187). In addition, the A/New York/1/18 strain of the 1918 pandemic possessed a Gly at position 222 and this markedly affected receptor binding, reducing α2-6 preference and increasing weak α2-3 [5].

**Figure:**
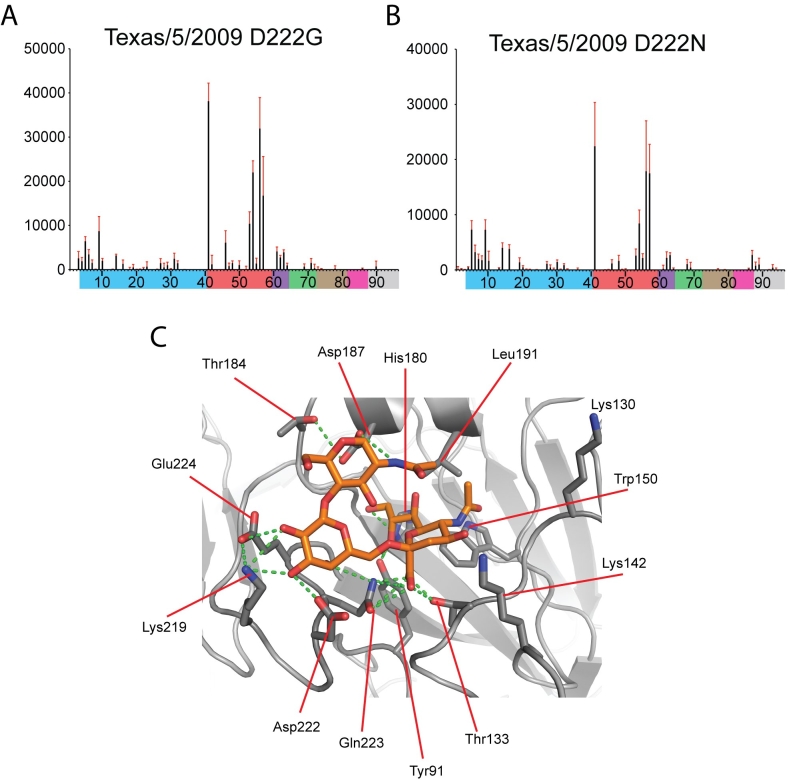
**Figure 3** Glycan microarray analysis of A/Texas/5/2009 mutants. The effect of position 222 mutations was assessed on the A/Texas/5/2009 framework by mutating the Asp to: A) a Gly and B) an Asn. Graphs are formatted as for Figure 2. C) The receptor binding site of Darwin09 with a 6’-sialyllactosamine (6’-SLN) modeled into the pocket highlights the residues that could contribute to the hydrogen bonding network between the receptor and the HA. Putative hydrogen bond interactions between the glycan and the HA RBS are shown as green broken lines.

        To address this question on the 2009 pandemic H1N1 virus, we mutated position 222 on the A/Texas/5/2009 HA to produce variants with either an Asp222Gly or an Asp222Asn mutation. Interestingly, glycan microarray analysis of these mutants revealed a α2-6 binding profile (Figure 3A and 3B) similar to the wild-type A/Texas/5/2009 recombinant HA (Figure 2). However, these mutants also bound weakly to sulfated α2-3 sialylglycans (glycans #4-8) as well as α2-3 and α2-3/α2-6 di-sialoside structures (glycans #9 & 10). Currently, it is unknown if the same profile will be reflected with viruses carrying the same mutations on the glycan microarray or if the increased valency of the virus due to the increased number of HAs on the virus surface will enhance this weak binding. Thus, on the current pandemic HA framework, the effect of these mutations at position 222 on receptor binding appears less dramatic when compared to the 1918 framework since the binding preference for α2-6 sialylglycans is still maintained. Analysis of the RBS of Darwin09 offers a possible reason. The galactose of α2-6-linked receptors can interact via its 3- and 2-hydroxyls through a hydrogen bond network using residues Lys219, Asp222 and Glu224. A loss of Asp222 through mutation might not compromise this network to the same extent as was seen in the 1918 HA framework when the Asp225Gly mutation was introduced [5].

## Conclusions

       Although a number of mutations have been reported in circulating pandemic H1N1 viruses, they have not affected virus antigenicity and pathogenicity. The use of the Darwin09 structure to analyze the interactions of these HAs with virus receptors highlights the importance of having structural information to aid such analysis. The expression system used here also provides an important route for the safe production of these pandemic proteins on a large scale. Availability of recombinant protein enables its use for downstream applications such as glycan microarray analysis, as described here, reagents for diagnostic kit development or as antigens for antibody production. If this methodology were not available, HA production from the virus would have been difficult at the start of the pandemic, due to stringent biosafety requirements.  Rapid determination and dissemination of the pandemic H1N1 hemagglutinin 3-D structure and characterization of its receptor specificity should enable the medical and public health research community to develop improved intervention approaches to control and prevent influenza morbidity and mortality as this virus becomes endemic in human populations. 

## Acknowledgements

        Use of the Advanced Photon Source at Argonne National Laboratory was supported by the U. S. Department of Energy, Office of Science, Office of Basic Energy Sciences, under Contract No. DE-AC02-06CH11357. The authors would like to thank the staff of SER-CAT sector 22 for their help with data collection and Ruben Donis (CDC) for help and advice during the project and preparation of this manuscript. The atomic coordinates and structure factors of the HA for Darwin/2001/2009 are available from the RCSB PDB under accession code 3M6S. Glycan microarray data presented here will be made available on-line through the Consortium for Functional Glycomics web site upon publication (www.functionalglycomics.org). The Glycan Microarray was produced for the Centers for Disease Control and Prevention using a glycan library generously provided by the Consortium for Functional Glycomics funded by National Institute of General Medical Sciences Grant GM62116. The findings and conclusions in this report are those of the authors and do not necessarily represent the views of the Centers for Disease Control and Prevention or the Agency for Toxic Substances and Disease Registry.

Funding Information

        Research was funded by the Centers for Disease Control and Prevention. 

Competing Interests

        "The authors have declared that no competing interests exist."
